# Neurocytological Advances in the Treatment of Glioblastoma Multiforme

**DOI:** 10.7759/cureus.16301

**Published:** 2021-07-10

**Authors:** Brian Fiani, Claudia Covarrubias, Chiduziem Onyedimma, Ryan Jarrah

**Affiliations:** 1 Neurosurgery, Desert Regional Medical Center, Palm Springs, USA; 2 School of Medicine, Universidad Anáhuac Querétaro, Santiago de Querétaro, MEX; 3 Neurological Surgery, Meharry Medical College, Nashville, USA; 4 Neurological Surgery, University of Michigan - Flint, Flint, USA

**Keywords:** regorafenib, paxalisib, glioblastoma multiforme, chemoagents, chemoradiotherapy, cannabinoids, immunotherapy, dianhydrogalactitol, selinexor, recombinant oncolytic poliovirus

## Abstract

Glioblastoma multiforme (GBM) is an aggressive neoplasm of the brain that has commonly led to disappointing patient outcomes. Despite medical advancements and increasing research efforts, GBM studies reveal a stagnant survival rate at the global level with only sluggish improvement over time. Modern neuro-oncology research places a heavy emphasis on pharmacological therapies. Through a broad database search, we accumulated and synthesized the GBM-related neuroimmunocytological literature to create a comprehensive and contemporary review. Based on our findings, we discuss the recent neurocytological treatment strategies for GMB and the results of the studies. Regorafenib, paxalisib, and dianhydrogalactitol (VAL-083) are showing initial promise to decrease disease progression. VAL-083 is an alkylating agent that creates N7 methylation on DNA and has the ability to cross the blood-brain barrier (BBB). Selinexor, recombinant nonpathogenic polio-rhinovirus, and GBM-vaccine of autologous fibroblasts retrovirally transfected with TFG-IL4-Neo-TK vector have all also shown initial clinical benefit in terms of prolonging survival. Most trials observe modest improvement in outcomes with a positive safety profile. Nevertheless, the need for further studies is warranted, along with the trending of post-therapeutic biomarkers in order to better access future patient outcomes.

## Introduction and background

Grade 4 astrocytomas, commonly referred to as glioblastoma multiforme (GBM) or malignant gliomas, are aggressive and fast-growing primary brain neoplasms [1]. GBMs are the most common tumors derived from glial cell origins and account for roughly 47.7% of all primary neoplasms, making them the most common type of primary brain tumors [2]. The incidence of these neoplasms is estimated to affect 3.21 persons per 100,000. It commonly affects middle-aged men more so than women, with epidemiological trends showing a higher occurrence among the white non-Hispanic population [1]. GBM's display dismal survival rates of approximately 40% in the first year following diagnosis [2]. 

There are several molecular and genetic aberrations that distinguish GBMs from other types of gliomas. These include the gain of chromosome 7p and a loss of chromosome 10q [3]. GBMs may be primarily or secondarily derived. Primary glioblastomas display no precursor lesions and frequently exhibit estimated glomerular filtration rate (eGFR) and mouse double minute 2 homolog (MDM2) amplification. Secondary GBMs are derived from lower-grade lesions and are often associated with isocitrate dehydrogenase-1 (IDH1) and O6-methylguanine-DNA methyltransferase (MGMT) mutations [4]. Histologically, these tumors consist of a heterogeneous mix of microcysts, pseudopalisading cells, are highly vascular, and contain areas of necrosis as a result of growth outstripping the blood supply [5]. In terms of treatment, the mainstay of management for GBMs is surgical resection, typically followed by radiation and chemotherapy. Gross total resection is of the utmost importance to prolong survival. The main focus of clinical trials in contemporary literature is centered on pharmacological approaches to slowing disease progression post-operatively. The increasing focus has been placed on targeted therapies for these neoplasms rather than systemic treatment regimens [6]. Advancements in GBM management have overall led to a slight increase in survival rates; however, the treatment of these aggressive tumors remains challenging. The length of survival from the time of diagnosis is exceedingly disappointing. Herein, we highlight the recent cytological advancements in the treatment of GMB and review the overall implications to survival longevity. We also assemble and synthesize the foundational clinical trials regarding neuroimmunocytological approaches to slowing disease progression.

## Review

Survival rate stagnation or progression?

Despite medical advances and the introduction of adjunct therapies, recent epidemiological studies have indicated a stagnant median survival rate at a global level as well as a rise in incidence, specifically in countries in South America, Eastern Europe, and Southern Europe [7, 8]. Between the years 2000 and 2014, the overall 1-year relative survival rate in the United States for patients diagnosed with GBM was estimated at 41.4% [9]. There was a marginal improvement from 34.4% (2000-2004) to 44.6% (2005-2014) [9]. In 2020, the Central Brain Tumor Registry of the United States (CBTRUS) published a statistical report accounting for the years 2013 to 2017, where the median observed survival in the primary malignant brain and other central nervous system (CNS) tumors was lowest for glioblastoma estimated at 8 months, whilst further emphasizing that the relative survival estimates at five years post-diagnosis remain low at 7.2% [8]. With the overall prognosis remaining poor and rare long-term survival, there is a need for precision therapies that can combat this aggressive disease that remains refractory to treatment [7, 10].

The World Health Organization (WHO) Classification of Tumors of the CNS was last revised in 2016 (fourth edition), with an upcoming 5th edition soon to be published [11, 12]. Although current histopathologic variants do not differ in treatment recommendations, the isocitrate dehydrogenase (IDH)-wildtype glioblastoma, corresponding to the clinically defined primary glioblastoma, is known to have a more aggressive clinical course [10, 12]. Additionally, a newly accepted variant was included in the fourth edition classification, the epithelioid glioblastoma, placed under the category of grade IV IDH-wildtype glioblastoma [12]. The unmethylated promoter status for MGMT is also being considered a validated biomarker for temozolomide (TMZ)-resistance, which further correlates with poor prognosis [13].

Traditional treatment methods

The optimal standard of treatment for newly diagnosed GBM includes maximal safe surgical resection, allowing for accurate histological diagnosis and tumor genotyping, followed by concurrent chemoradiotherapy (CCRT) [10, 14-16]. If feasible, the surgical adjunct of intraoperative fluorescence-guided surgery using 5-aminolevulinic acid (5-ALA) with a gross total resection (GTR) surgical approach is generally recommended, with additional intraoperative imaging guidance [10, 11]. The current standard of treatment was published as the Stupp protocol in 2005, which has since demonstrated clinically meaningful and statistically significant survival benefit as well as minimization of additional toxicity [14-16]. TMZ, the primary FDA-approved drug used to treat GBM, is an orally administered DNA alkylating agent that effectively crosses the blood-brain barrier (BBB) and has previously demonstrated anti-tumor efficacy [14, 16, 17]. CCRT is usually initiated four weeks post-surgery for a duration of 6 weeks, followed by adjuvant chemotherapy with TMZ for 6 months [18]. The use of radiotherapy (RT) has also been well established, remaining as an important component in the management of GBM [19]. Currently, the use of stereotactic radiosurgery, as well as short-course hypo-fractionated stereotactic RT, is being studied in order to further explore feasibility and safety [19].

Apart from the chemo agent TMZ, other FDA-approved GBM therapies include lomustine, carmustine, bevacizumab, intracavitary carmustine wafers, and tumor treating fields (TTFs) [18, 20]. The introduction of concurrent management modalities has prolonged survival times in patients with GBM [10]. TTFs is a GBM treatment therapy with low-intensity, alternating electric fields are delivered by transducer arrays applied to the shaved scalp [10, 11, 21]. TTFs given during maintenance TMZ have demonstrated progression-free survival of 6.7 months versus TMZ alone [10, 21]. Furthermore, the management of recurrent GBM (rGBM) remains a challenge as there is no established standard of care, with an individualized approach, with supportive and palliative care playing a significant role throughout the disease progression. Systemic therapies for rGBM include nitrosourea, lomustine, or the antiangiogenic therapy with bevacizumab, a humanized monoclonal antibody that inhibits vascular endothelial growth factor (VEGF) [10, 19]. The overall quality of data for individual chemotherapy agents continues to portend poor outcomes, and it is generally recommended that patients with both newly diagnosed GBM, as well as rGBM, enroll in clinical trials that assess the safety and tolerability of novel therapeutics [19, 21]. Although the optimal dose-fractionation regimen for re-irradiation remains unclear, it has been found to have reasonable efficacy with an acceptable safety profile in selected patients with rGBM [21].

Novel treatment methods

There are several international randomized controlled studies that have failed to significantly demonstrate the survival advantage with immunotherapy and precision oncology approaches [10, 22]. Notably, the BBB and the unique immune microenvironment have represented a challenge in the development of successful novel therapies that can demonstrate better efficacy alongside lower toxicity [10]. The GBM adaptive, global, innovative learning environment (GBM AGILE) is an ongoing international response adaptive randomization platform trial designed to evaluate multiple therapies in newly diagnosed (ND) and rGBM with three experimental arms being treated with regorafenib, paxalisib, and VAL-083, respectively [23]. Actively studied VAL-083, also known as dianhydrogalactitol, is an alkylating agent that creates N7 methylation on DNA and has the ability to cross the BBB [13, 23].

Abnormalities in epidermal growth factor receptor (EGFR) are common, with a 30% to 60% rate of aberrancy in glioblastomas alone [22]. A recent Cochrane publication conducted by Lee et al. concluded that there is insufficient evidence that demonstrates overall survival benefit with the addition of anti-EGFR therapy in first-line and recurrent glioblastomas [22]. From other therapeutic perspectives, the investigation of cannabinoids in oncology has increased significantly in recent years [15]. Although there is still limited understanding of their anti-cancer effects, there have been multiple preclinical models that support the role of modulation of cannabinoids in affecting tumorigenesis as well as tumor microenvironment [14]. Cannabinoid receptors CB1 and CB2 are both expressed in gliomas with heterogeneous immunohistochemistry levels [15]. Doherty et al. recently published a study analyzing the feasibility of nabiximols oromucosal spray, a formulation of both tetrahydrocannabinol (THC) and cannabidiol (CBD) in an ~ 1:1 ratio, given in combination with dose-intense temozolomide (DIT), which yielded unsatisfactory results in the GBM population [15].

Selinexor (SEL) is a novel treatment and the first oral selective inhibitor of a nuclear export compound that is tested for cancer treatment [24, 25]. A phase 1 interventional study evaluating the safety and tolerability of SEL for advanced or metastatic solid tumor malignancies was published in 2017 where the authors reported 100% of GBM experimental arm participants with Treatment Emergent Adverse Events (TEAEs) and an average of 41 days of progression-free survival (PFS) [25]. Additionally, there is an ongoing phase 1 clinical trial (clinicaltrials.gov; NCT04216329) assessing the safety, tolerability, and maximum tolerated dose of SEL in combination with TMZ and external beam RT in patients with newly diagnosed GBM [26].

Other promising novel therapies in the treatment of GBM currently being studied include the recombinant oncolytic poliovirus. A randomized controlled trial (RCT) studied the recombinant nonpathogenic polio-rhinovirus chimera (PVSRIPO) with the goal of estimating the efficacy of the anti-tumoral virus and the optimal dose [27]. Furthermore, peptide-based and dendritic cell vaccination have also undergone phase II and phase III RCTs [28].

Trial outcomes

Over the past three decades, clinical trials have expanded to investigate novel therapeutic regimens with the goal to improve GBM patient outcomes. There have been promising phase I & II trials investigating immunotherapy as a potential treatment modality [29]. Brada et al. describe one open-label, uncontrolled, multicenter phase II trial where TMZ was used for 138 chemotherapy-naive patients with GBM after the first relapse of GBM [30]. The results showed that PFS after six months was 18% for the selected population, with 45% describing a stabilized pathological course. The results of this trial were modest yet showed certain quality improvement in the lives of patients and a favorable safety profile [30]. In another study by Okada et al., two patients received a GBM-vaccine of autologous fibroblasts retrovirally transfected with TFG-IL4-Neo-TK vector [31]. The study showed both patients having a positive clinical response, with biopsies from the vaccine site showing immunological responses of IL-4 dependent infiltration of CD4+ and CD8+ T-cells. The enzyme-linked immune absorbent spot (ELISpot) assays showed systemic T-cell responses against an HLA-A2-restricted glioma-associated antigen (GAA) [31]. Additionally, both participants showed clinical and radiological improvement with no adverse events of encephalitis; however, tumor recurrence did eventually manifest for both patients [31]. Nevertheless, the treatment was well received, and further studies would provide greater insight into the efficacy of this potential therapy. In another study by Crane et al., a cancer immunotherapy vaccine treated with 96 kD chaperone proteins from brain tissue containing GBM was immunomonitored in 12 patients [32]. Neurocytological testing of peripheral blood leukocytes before and after vaccination showed a significant peripheral immune response for the HSP-96 peptide in 11 treated patients [32]. Brain biopsies of immune responders showed considerable CD4, CD8, and CD56 positive cell infiltrates within the immune responders. No adverse events attributed to the vaccine, with the study showing the first evidence for a human immune response against autologous tumor-derived peptides [32]. In a study by Liau et al., a phase 1 trial was established to evaluate 12 GBM patients treated with dendritic cell vaccines [33]. Results showed that six patients had measurable systemic anti-tumor responses, with one patient showing an objective clinical response verified through MRI imaging [33]. However, the vaccine did not translate into any increased survival (Table 1) [33].

**Table 1:** Overview of previous human clinical trials assessing various therapeutic approaches for the treatment of GBM GBM - glioblastoma multiforme

Author	Key Finding
Brada et al. [30], 2001	Temozolomide showed modest clinical efficacy with a positive safety profile. Improvement in quality of life was measured in patients with recurrent GBM.
Liau et al. [33], 2005	Dendritic cell vaccination in GBM patients showed some measured improvements, however, this did not necessarily translate into increased survival.
Okada et al. [31], 2007	Autologous glioma cell vaccine showed clinical and radiological improvement yet both GBM patients in the study eventually had tumor recurrence.
Crane et al. [32], 2013	Peptides bound to a 96 kD chaperone protein (HSP-96) from brain tissue containing GBM can be used to safely immunize patients with recurrent GBM.
Roa et al. [34], 2015	There were no differences in overall survival time between short-course radiotherapy and the conventional course for radiotherapy.
Perry et al. [35], 2017	Patients with radiotherapy with concomitant and adjuvant temozolomide had longer survival times than short-course radiotherapy alone.

In trials that assess radiotherapy, Roa et al. conducted a study that focused on observing the optimal radiotherapy dose regimen. In the study, 98 elderly GBM patients were randomized into two trial groups [34]. The first group received a short-course radiotherapy, while the second received the commonly used radiotherapy dosage. The results showed no statistical or observable difference between the two groups when assessing for quality of life, progression-free survival time, and overall survival time. This showed that short-course radiotherapy can be a potential therapeutic option for elderly cases of GBM [34]. In a similar trial by Perry et al., 562 elderly patients were randomized to receive radiotherapy alone (Group A) or radiotherapy with concomitant and adjuvant TMZ (Group B) [35]. The median overall survival time was observed to be higher for Group B than Group A (9.3 months vs. 7.6 months), indicating that the addition of TMZ to short-course radiotherapy leads to better survival outcomes of elderly patients with GBM [35].

Immune checkpoint inhibition studies investigate inhibitory receptors on T-cells, including CTLA-4 and PD-1 [36]. Glioma rodent models provide some evidence that blockage of CTLA-4 and PD-1 can promote long-term survival [36]. Therefore, ongoing trials are investigating immune checkpoint inhibition studies as a potential treatment. Moreover, other immunotherapy trials are emphasizing a focus on vaccines and antibody therapy regimens [36]. Nevertheless, GBM trials present limitations and challenges that have warranted inventive approaches ranging from gene therapy to combinatorial immunotherapeutic treatment models. Current obstacles for GBM therapy include finding drugs that can penetrate the BBB, identifying specific tumor antigens, establishing therapeutic biomarkers, while also augmenting vaccine therapies and preserving safety outcomes [36].

## Conclusions

The treatment of GBM requires a contemporary approach with both traditional and novel treatment models to provide improved strategies for optimizing patient outcomes. While ongoing trials are showing some promise, future studies are warranted to better address GBM tumors and prolong patient survival. The established evidence for post-therapeutic immunological biomarker identification and monitoring is clear. Trending neuroimmunological and neurocytological markers can be useful for indicating therapeutic success and can enhance the framework of ongoing trials. Pharmacological methods to supplement current treatment methods will be the true test of time. In conclusion, GBM clinical trials to date provide only modest evidence of prolonging survival.
